# The Genetic Diversity and Structure of the European Turtle Dove *Streptopelia turtur*

**DOI:** 10.3390/ani11051283

**Published:** 2021-04-29

**Authors:** Petras Prakas, Dalius Butkauskas, Saulius Švažas, Antonio Bea, Vadym Yanenko, Adomas Ragauskas, Daiva Vaitkuvienė

**Affiliations:** 1 Nature Research Centre, Akademijos Str. 2, LT-08412 Vilnius, Lithuania; dalius.butkauskas@gamtc.lt (D.B.); saulius.svazas@gamtc.lt (S.Š.); adomas.ragauskas@gamtc.lt (A.R.); daiva.vaitkuviene@gamtc.lt (D.V.); 2 Ekos Estudios Ambientales S.L.U., Donostia Etorbidea, 2 Bajo. Local 2, 20160 Lasarte, Spain; a.bea@ekos-sl.com; 3 National Museum of Nature History of Academy of Sciences of Ukraine, 15 B. Khmelnitsky Str., 01030 Kyiv, Ukraine; ornithologist.ua@gmail.com

**Keywords:** *Streptopelia turtur*, mtDNA, genetic variability, genetic structure, conservation

## Abstract

**Simple Summary:**

The European Turtle Dove, *Streptopelia turtur*, is a widespread Palearctic species. Due to a long-term population decline, it is listed as vulnerable by the IUCN. Population genetics studies are important to the management of threatened species. Previous research based on mitochondrial DNA cytochrome-b of European Turtle Doves sampled in Western and Southern Europe showed a lack of genetic structure of this species. The present study aimed to identify the possible genetic divergence in the European Turtle Dove. A total of 258 birds collected from Spain, Ukraine, and Morocco were examined using mitochondrial DNA cytochrome-b and D-loop sequence analysis. The high genetic diversity was evaluated in both loci analysed. Various population genetic analyses displayed genetic differences between Turtle Doves from Morocco and Ukraine, and certain Spanish samples. The results of this study will be vital for effective conservation and sustainable management of this vulnerable species.

**Abstract:**

The European Turtle Dove, *Streptopelia turtur*, a long-distance migrant wintering in Africa, is a widespread Palearctic species. This species is classified as vulnerable and is undergoing a long-term demographic decline. The results of the previous study (based on mitochondrial (mtDNA) cytochrome-b (*cytb*) sequences of birds from Western and Southern Europe) indicated that the species was not genetically structured. We analysed the mtDNA *cytb* and D-loop of 258 birds collected from Morocco, Spain, and Ukraine. High genetic variability, expressed by haplotype diversity and nucleotide diversity, was revealed in both *cytb* (Hd = 0.905 ± 0.009, π = 0.00628 ± 0.00014) and the D-loop (Hd = 0.937 ± 0.009, π = 0.01502 ± 0.00034). SAMOVA and principal coordinates analysis revealed the birds belonged to two genetically distinct groups. One group included birds collected in Spain, while birds sampled in Morocco and Ukraine formed another group. Furthermore, significant genetic differentiation was identified between Turtle Doves from Morocco and Ukraine, and certain Spanish samples. The present results indicate that specific management and conservation plans relevant for the species in various regions should be applied. However, further nuclear DNA research and new studies (particularly in Eastern Europe) are necessary for the decisive results on genetic structure of this species.

## 1. Introduction

The European Turtle Dove, *Streptopelia turtur* (hereafter, Turtle Dove), is a widespread Palearctic species [1,2]. The European population is estimated at approximately 2.9 to 5.6 million pairs [3]. It is a long-distance migrant and its post-breeding migration towards Africa reaches its most intensive period in August–September [1]. Several flyways connecting the breeding grounds in Europe and the wintering areas in Africa have been designated. The western migratory route stretches across the Iberian Peninsula and Morocco, while other routes pass through Italy, Malta, Tunisia, and through the Balkan countries, Egypt, and the Middle East [4]. Turtle Doves breeding in European Russia and Ukraine migrate mainly to Eastern Africa via Turkey and the Middle East [5]. Since 2015 it has been classified as Vulnerable by the IUCN because of a long-term population decline [6]. In Europe, the population size is estimated to have decreased by 30–50% over 16 years (three generations) [6]. The nominal subspecies, *Streptopelia turtur turtur*, is listed in Appendix II of the Convention on Migratory Species.

In this context, it is essential to study the population genetic structure of the Turtle Dove. Yet only one study analysing genetic population structure of this species has been conducted [7]. Previous genetic studies of the Columbidae family of birds primarily focused on phylogeny reconstruction [8,9,10]. Also, intraspecific genetic variation of the Feral Pigeon *Columba livia*, was extensively examined [11,12,13,14]. Meanwhile, comprehensive studies analysing genetic structure and diversity of other species of the Columbidae family are still scarce [15].

Results from the earlier research based on the cytochrome-b (*cytb*) sequences and nuclear SNP analysis of Turtle Doves, though limited to Western and Southern Europe, suggested that the species is panmictic across Europe [7]. Therefore, similar conservation actions across Europe were recommended in the International Single Species Action Plan for the Conservation of the European Turtle Dove [3]. However, if the species is genetically structured across flyways, different threats and conservation actions can be relevant between separate regions and populations. Identification of intraspecific evolutionary significant units is crucial for the long-term conservation of threatened bird species [16,17].

The aim of this study was to identify possible genetic divergence of the Turtle Dove based on *cytb* and D-loop analyses of birds sampled in Ukraine, Spain, and Morocco. The research includes two subspecies: the nominal *Streptopelia turtur turtur* breeding in continental Europe and *S*. *t*. *arenicola* breeding in Morocco and the Balearic Islands [18,19].

## 2. Materials and Methods

### 2.1. Sample Collection

Samples from 258 Turtle Doves were used for genetic analysis. Blood samples were taken from the brachial vein of live individuals in their breeding sites in Morocco in 2018–2019. Tissue samples were taken from the heart or liver of legally hunted individuals in Spain, Ukraine, and Morocco in 2017–2020. Birds in Spain (possibly including both local birds and migrants from other parts of Europe) were collected from six sites: in the Balearic Islands (*n* = 23), in Eastern Spain (Catalonia, *n* = 25), in Central Spain (Almaraz site, *n* = 35 and Pereleda de Roman site, *n* = 28) in Southern Spain (Palma del Rio site, *n* = 20) and at the Strait of Gibraltar coast of Spain (Vejer de Frontera site, *n* = 31). Birds migrating and breeding in Morocco were sampled in Southwestern Morocco (Taroudant site, *n* = 25), while in central Morocco—only local breeding birds were sampled (Beni Mellal site, *n* = 29). Migrating birds were sampled in Central Ukraine (*n* = 42).

### 2.2. DNA Isolation, Amplification and Sequencing

Genomic DNA was extracted following the universal and rapid salt-extraction method [20] and eluted in 300 μL of nuclease-free water. The partial mitochondrial DNA (mtDNA) D-loop region was amplified using PAL-2 forward (5′-CATATTCATGACCCCCATACG-3′) and reverse (5′-GGCCTGAAGCTAGTCGTGAT-3′) primers [21], yielding a 440 bp long fragment. F_cytb_St (5′-TGATAACTCAAATCCTAACTGGTC-3′) and R_cytb_St (5′-TTGTTTTCTAGGGCTCCGAT-3′) were used to amplify partial, 1003 bp long mtDNA *cytb* [7]. The total volume of each PCR mixture was 25 μL, containing 12.5 μL of Dream Taq PCR Master Mix (Thermo Fisher Scientific, Vilnius, Lithuania), 0.2 μM of each primer, 100 ng template DNA, and nuclease-free water. For the D-loop, amplification started with initial denaturation for 2 min at 95 °C, followed by 35 cycles of denaturation at 94 °C for 30 s, annealing at 58 °C for 45 s and elongation at 72 °C for 45 s, and finishing with a final extension at 72 °C for 5 min. For *cytb*, amplification proceeded via an initial hot start at 95 °C for 5 min followed by 35 cycles of 94 °C for 45 s, 60 °C for 60 s, 72 °C for 70 s, and a final extension at 72 °C for 7 min. The PCR products were evaluated using a 1.5% agarose gel and purified with exonuclease ExoI and alkaline phosphatase FastAP (Thermo Fisher Scientific, Vilnius, Lithuania). PCR products were sequenced directly using the same forward and reverse primers as for amplification. The Big-Dye® Terminator v. 3.1 Cycle Sequencing Kit and the 3500 Genetic Analyser (Applied Biosystems, Foster City, CA, USA) were used for performing sequencing reactions. The D-loop and *cytb* sequences generated in the present study were deposited in GenBank under accession numbers MW438351–MW438608 and MW438609–MW438866, respectively.

### 2.3. Data Analysis

The *cytb* sequences obtained in our study were compared with 94 sequences (KU588290-KU588313, KU588315-KU588384) obtained by Calderón et al. [7] which included Turtle Dove samples from Spain, the UK, France, Italy, Malta, Bulgaria, and Greece. The D-loop and *cytb* sequences were aligned using the MUSCLE algorithm implemented into the MEGA7 [22]. The beginning and the end of some sequences were truncated to have all sequences beginning and ending at the same nucleotide positions. FaBox v. 1.5 was used to identify different haplotypes [23]. The selection of nucleotide substitution model and phylogenetic analysis of identified haplotypes was carried out with MEGA7 using the maximum likelihood method. Tamura-Nei + G + I, and HKY + G evolutionary models were set for D-loop and *cytb*, respectively. The bootstrap phylogeny test was performed with 1000 replicates. DnaSP v 6 was used for calculation of some intraspecific genetic variation measurements, the number of haplotypes (h), the number of variable sites (S), parsimony informative sites, haplotype diversity (Hd), and nucleotide diversity (π) [24].

Genetic differentiation for the Turtle Dove sample pairs was evaluated using Φ_ST_ with Arlequin v. 3.5.2.2 [25]. The statistical significance of each pairwise Φ_ST_ was tested by 10,000 permutations at the 95% confidence level.

We assessed population structure using spatial analysis of molecular variance with SAMOVA v. 2.0 [26]. We chose the group of populations (K) value which maximised among group genetic variation (Φ_CT_) and was significant (*p* < 0.05). Principal coordinates analysis (PCoA) based on Nei’s [27] distance was conducted in GenAlEx v. 6.502 [28].

## 3. Results

### 3.1. Genetic Variation

**Figure 1:**
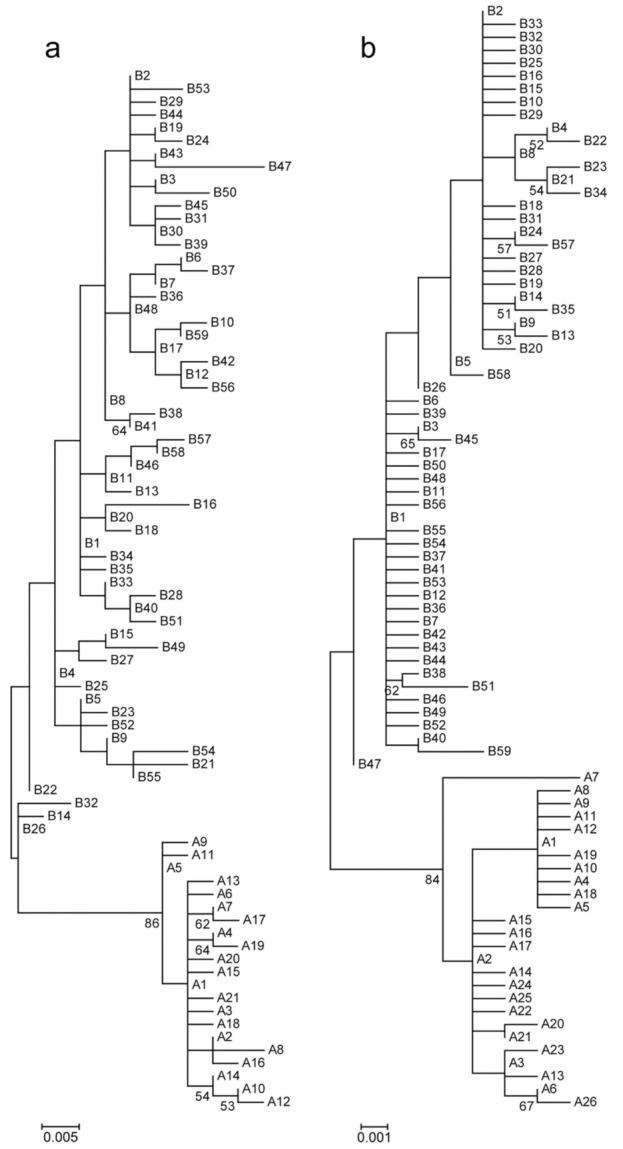
Midpoint rooted maximum likelihood phylogenetic trees of the Turtle Dove based on D-loop (**a**) and *cytB* (**b**) haplotypes. Figures show bootstrap support values higher than 50%. GenBank accession numbers are indicated for those *cytb* haplotypes identified only by Calderon et al. [7]. A10 = KU588369, A11 = KU588323, A12 = KU588309, A19 = KU588313, A24 = KU588354, A25 = KU588338, A26 = KU588372, B12 = KU588322, KU588360, KU588362, B16 = KU588311, KU588324, B17 = KU588332, KU588378, B21 = KU588300, B26 = KU588351, B30 = KU588367, B31 = KU588355, B32 = KU588344, B33 = KU588310, B34 = KU588305, B38 = KU588320, B43 = KU588358, B44 = KU588341, B53 = KU588376, B54 = KU588371, B55 = KU588307, B56 = KU588304, B60 = KU588301.

D-loop analysis was conducted on samples from 258 Turtle Doves collected in nine sites in different regions of Spain, Morocco, and Ukraine. In 371-bp sequences, we obtained 44 variable sites, of which 28 were parsimony informative. Eighty haplotypes were identified. The mean frequency of haplotypes was 3.2. Of 80 haplotypes, 48 (60.0%) were singletons. The frequency of 27 haplotypes ranged from 2 to 7, the frequency of four haplotypes ranged from 11 to 23, and the most common haplotype (A1) was confirmed in 52 individuals. The high bootstrapping support value (86) was given to divide haplotypes into two clusters (Figure 1a). Twenty-one haplotypes were assigned to haplogroup “A”, and the remaining 59 haplotypes were assigned to haplogroup “B”. The latter haplogroup included 65.1% of the total sample. Haplogroup “B” was characterised not only by a higher abundance of haplotypes but also by a higher haplotype diversity (Hd = 0.948 ± 0.008) and nucleotide diversity (π = 0.00722 ± 0.00032) than haplogroup “A” (Hd = 0.659 ± 0.057, π = 00.00286 ± 0.00039; Table 1).

We identified 68 *cytb* haplotypes from the analysis of 258 Turtle Doves. The *cytb* sequences generated in the present study were compared with sequences obtained by Calderón et al. [7]. The final *cytb* analysis included 352 sequences and identified 93 haplotypes. Of 892 nucleotide positions, 76 were polymorphic, 36 of which were parsimony informative sites. The mean frequency of haplotypes was 3.8. Of the 93 haplotypes, 66 were present in a single individual (71.0%), 22 haplotypes were represented by 2–7 individuals, and the five most common haplotypes (A1, A2, A3, B1, and B2) were represented by 13, 21, 50, 60, and 71 individuals, respectively. The three most frequent haplotypes comprised 51.4% of the total sample. As in the case of D-loop, *cytb* haplotypes clustered into two groups (Figure 1b): 30 haplotypes were attributed to haplogroup “A”, while 63 were attributed to haplogroup “B”. Overall, 63.6% of the sample was assigned to haplogroup “B”. Unlike the D-loop data, the *cytb* sequence analysis demonstrated similar haplotype diversity and nucleotide diversity values in both haplogroups (Table 1). *Cytb* and D-loop analyses assigned samples from all individuals using both mtDNA loci to the same haplogroup. High haplotype diversity values were identified for both *cytb* (Hd = 0.905 ± 0.009) and the D-loop (Hd = 0.937 ± 0.009). More than twice, the D-loop showed higher total nucleotide diversity (π = 0.01502 ± 0.00034) than *cytb* (π = 0.00628 ± 0.00014).

### 3.2. Population Genetic Structure

**Figure 2:**
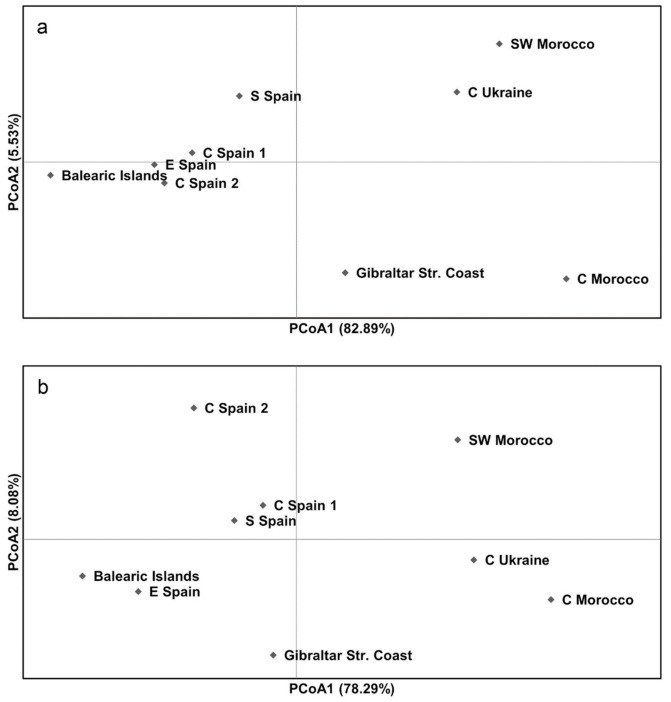
Principal coordinates analysis (PCoA) of the Turtle Dove samples based on D-loop (**a**) and *cytb* (**b**) sequences.

Based on D-loop and *cytb* results, the detected pairwise Φ_ST_ values showed a genetic divergence between birds collected in Spain and birds collected in Morocco and Ukraine (Table 2). Significant differentiation was found at both loci between birds collected in Morocco and Ukraine, and birds collected in the Balearic Islands and eastern Spain. The highest Φ_ST_ values were observed between Turtle Doves from central Morocco and Spanish samples. Based on SAMOVA analysis, the highest genetic differentiation among group values occurred when combining examined samples into two groups, both for D-loop (Φ_CT_ = 0.079, *p* < 0.01) and *cytb* (Φ_CT_ = 0.103, *p *< 0.01) data. At both loci, Morocco and Ukraine samples formed one group, while Spanish samples formed another group. The principal coordinates analysis (PCoA) demonstrated a close relationship between Turtle Doves sampled in Ukraine and Morocco (Figure 2).

## 4. Discussion

### 4.1. Population Genetic Studies in Columbidae

Examination of population genetics in Columbidae species is mainly based on microsatellite markers and maternal mtDNA sequence analysis. Microsatellite markers have been used to determine the genetic diversity, gene flow, and relationships between different populations or lineages [11,13,29,30], while mtDNA has been applied for the analysis of genetic variability, genetic structure, and phylogeography of different Columbidae species [15,31]. For population genetic analysis of Columbidae species, different regions of mtDNA, the noncoding D-loop, *cytb*, and cytochrome oxidase I (*COI*) were used [7,15,17,30,31,32,33]. To date, there was only one study on the genetic structure of the Turtle Dove based on the results of *cytb* analysis, with samples analysed from 95 birds collected in eight countries of Western and Southern Europe (1–17 individuals sampled in each country) [7]. In our study, 258 Turtle Doves collected in Ukraine (representing the flyway used by birds breeding in Eastern Europe), Spain (representing the western flyway), and Morocco (representing subspecies *S. t. arenicola*), based on *cytb* and D-loop sequences, were examined. The obtained genetic differences between certain Turtle Dove populations have revealed the necessity of appropriate management and conservation measures for this threatened species.

### 4.2. Genetic Diversity in the Turtle Dove

A high genetic variability expressed in the number of polymorphic sites (S), the number of haplotypes (h), the haplotype diversity (Hd), and the nucleotide diversity (π) were assessed at both studied mtDNA loci (Table 1). Based on D-loop analysis, nucleotide diversity obtained in the Turtle Dove (π = 0.01502 ± 0.00034) was significantly higher than that observed for the Japanese Woodpigeon *Columba janthina* (π = 0.0009–0.0057 [31], π = 0.001049 ± 0.001015 [17]) and the New Zealand Pigeon *Hemiphaga novaeseelandiae* (π = 0.00142 ± 0.00036) [32]. Both species are endemic with a limited distribution range in specific regions. In contrast, at the same loci, significantly lower nucleotide diversity was determined for the Turtle Dove than for the Woodpigeon *Columba palumbus* (π = 0.04113 ± 0.00150), which is an abundant and widespread Palearctic species [33]. The European population of the Turtle Dove has undergone a marked demographic decline [7], which probably caused a reduction of the intraspecific variability of the species.

### 4.3. Evolutionary Lineages of Turtle Doves

**Figure 3:**
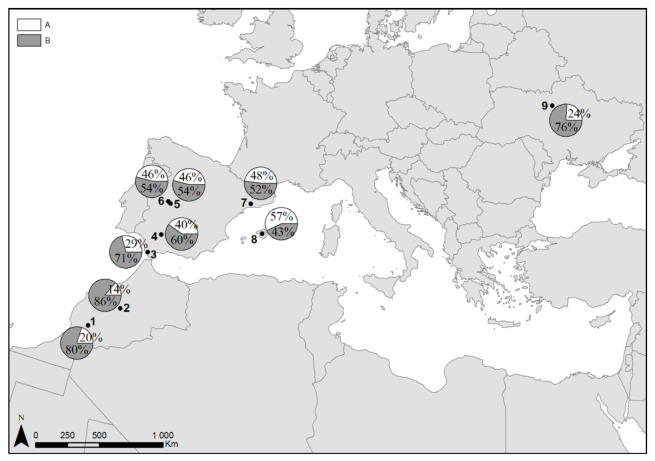
Distribution of two haplogroups (A and B) defined in mtDNA *cytb* and D-loop for the Turtle Dove populations. 1—Southwestern Morocco; 2—Central Morocco; 3—Strait of Gibraltar coast; 4—Southern Spain; 5—Central Spain 1; 6—Central Spain 2; 7—Eastern Spain; 8—the Balearic Islands; 9—Central Ukraine.

This study identified two evolutionary lineages in both analysed mtDNA regions: the D-loop and *cytb* (Figure 1). The existence of two main haplogroups within the *cytb* of the Turtle Dove has been previously suggested, and the median-joining network demonstrated that they were differentiated by six mutational steps [7]. The ratio of haplogroups “A” and “B” was different across geographic regions (Figure 3). The frequency of the haplogroup “A” increased from Morocco to Eastern Spain. In birds sampled in Morocco the frequency of the haplogroup “A” ranged from 14 to 20%. The haplogroup “A” ratio increased from 29% in birds sampled at the coast of the Strait of Gibraltar in Spain to 48% in eastern Spain. The highest ratio of the haplogroup “A” (57%) was observed in Turtle Doves collected in the Balearic Islands. In contrast, low frequency of the haplogroup “A” (24%), similar to that detected in birds of subspecies *S. t. arenicola* in Morocco, was identified in birds of the nominal subspecies migrating via Ukraine. It is possible that Turtle Doves of the nominal subspecies breeding in Eastern Europe mix with *S. t. arenicola* on the wintering grounds [34]. Notably, two genetic groups within the mtDNA *COI* were also shown for the Eurasian Collared Dove *Streptopelia decaocto* [15]. The genetic segregation of the species can be possibly caused by the former geographic isolation of different populations [7]. Although subspecies *Streptopelia t. turtur* and *Streptopelia t. arenicola* differ morphologically, significant genetic differences between them were not observed within mtDNA, which indicated the recent evolutionary origin, as it was earlier suggested for different subspecies of the Dunlin *Calidris alpina* [35].

### 4.4. Species Management and Conservation Implications

Genetic analysis based on mtDNA sequencing data is increasingly used to designate conservation priorities and flyways of bird species [33,36,37]. The previous study of the mtDNA *cytb* sequence analysis showed no evidence of population genetic structure of Turtle Doves sampled in Western and Southern Europe [7]. By contrast, we identified significant genetic differentiation between birds collected in Morocco and Ukraine, and birds collected in certain regions of Spain (Table 2). Additionally, principal coordinates analysis showed a close genetic relationship between Morocco and Ukrainian samples, and their differences from Spanish samples (Figure 2). Therefore, the results of our study indicate that the European population of the Turtle Dove is genetically structured within mtDNA. These results are essential for the long-term sustainable management of this vulnerable species. The genetic differences identified for the Turtle Dove require an appropriately revised International Single Species Action Plan for the Conservation of the European Turtle Dove (2018–2028), as different conservation and management priorities can be applied in specific regions for populations with variable genetic structure. Further detailed research of the Turtle Dove is particularly important in Eastern Europe due to distinct genetic structure suggested for birds from this region.

## 5. Conclusions

Based on mtDNA D-loop and *cytb* sequences, high genetic variability was shown for the Turtle Dove, a vulnerable species experiencing a long-term population decline. The genetic differences among birds sampled in Morocco, Ukraine, and Spain were observed. For conclusive results on the Turtle Dove’s population genetic structure, further research covering a representative sample from different regions of Europe and the use of nuclear DNA markers are necessary.

## Figures and Tables

**Table 1:** Estimates of intra-population genetic variability of mtDNA D-loop and *cytb* sequences of the Turtle Dove, *Streptopelia turtur*.

Sample	*n*	S	h	Hd ± SD	π ± SD
D-loop					
Taroudant (Southwestern Morocco)	25	20	17	0.950 ± 0.029	0.01443 ± 0.00201
Beni Mellal (Central Morocco)	29	20	21	0.978 ± 0.014	0.01234 ± 0.00184
Vejer de Frontera (Strait of Gibraltar coast)	31	15	17	0.931 ± 0.029	0.01331 ± 0.00110
Palma del Rio (Southern Spain)	20	14	9	0.832 ± 0.063	0.01461 ± 0.00094
Almaraz (Central Spain 1)	35	22	15	0.909 ± 0.027	0.01615 ± 0.00085
Pereleda de Roman (Central Spain 2)	28	20	17	0.950 ± 0.024	0.01612 ± 0.00081
Catalonia (Eastern Spain)	25	23	18	0.927 ± 0.045	0.01655 ± 0.00101
Balearic Islands (Spain)	23	13	13	0.877 ± 0.061	0.01387 ± 0.00119
Central Ukraine	42	18	23	0.950 ± 0.018	0.01364 ± 0.00138
A haplogroup	90	20	21	0.659 ± 0.057	0.00286 ± 0.00039
B haplogroup	168	59	32	0.948 ± 0.008	0.00722 ± 0.00032
Overall	258	44	80	0.937 ± 0.009	0.01502 ± 0.00034
*cytb*					
Taroudant (Southwestern Morocco)	25	22	12	0.797 ± 0.077	0.00486 ± 0.00094
Beni Mellal (Central Morocco)	29	24	19	0.958 ± 0.021	0.00520 ± 0.00078
Vejer de Frontera (Strait of Gibraltar coast)	31	17	10	0.854 ± 0.035	0.00583 ± 0.00056
Palma del Rio (Southern Spain)	20	20	12	0.921 ± 0.042	0.00696 ± 0.00068
Almaraz (Central Spain 1)	35	21	14	0.859 ± 0.038	0.00623 ± 0.00037
Pereleda de Roman (Central Spain 2)	28	21	12	0.905 ± 0.030	0.00639 ± 0.00043
Catalonia (Eastern Spain)	25	18	11	0.883 ± 0.042	0.00656 ± 0.00040
Balearic Islands (Spain)	23	20	11	0.877 ± 0.049	0.00650 ± 0.00065
Central Ukraine	42	23	17	0.900 ± 0.028	0.00521 ± 0.00053
Comino * (Malta)	8	15	8	1.000 ± 0.063	0.00637 ± 0.00128
Monfrague * (Central Spain)	10	16	10	1.000 ± 0.045	0.00717 ± 0.00093
Dobrich * (Bulgaria)	14	14	7	0.879 ± 0.058	0.00623 ± 0.00077
Essex, Norfolk * (UK)	14	15	7	0.857 ± 0.065	0.00591 ± 0.00067
Pitou-Charente, Auvergne, Marne * (France)	15	21	13	0.981 ± 0.031	0.00728 ± 0.00101
Evros * (Greece)	16	19	11	0.950 ± 0.036	0.00656 ± 0.00073
Isla Ventotene * (Italy)	17	17	9	0.875 ± 0.058	0.00719 ± 0.00051
A haplogroup	128	30	30	0.745 ± 0.036	0.00215 ± 0.00017
B haplogroup	224	51	63	0.848 ± 0.018	0.00277 ± 0.00010
Overall	352	76	93	0.905 ± 0.009	0.00628 ± 0.00014

*n*—sample size, S—number of variable sites, h—number of haplotypes, Hd—the haplotype diversity, π—the nucleotide diversity, SD—standard deviation, * data from Calderón et al. [7].

**Table 2:** Genetic differentiation for the Turtle Dove population pairs. Pairwise Φ_CT_ obtained based on D-loop and *cytb* sequences are shown below and above the diagonal, respectively. Values in bold are statistically significant (*p* < 0.05).

	1	2	3	4	5	6	7	8	9
1 Southwestern Morocco		0.013	0.037	0.036	0.029	**0.072**	**0.106**	**0.152**	0.007
2 Central Morocco	−0.008		**0.095**	**0.113**	**0.105**	**0.158**	**0.179**	**0.234**	−0.002
3 Strait of Gibraltar coast	0.012	0.027		−0.018	−0.014	0.016	−0.001	0.023	**0.062**
4 Southern Spain	0.033	**0.085**	−0.012		−0.034	−0.015	−0.018	0.001	**0.074**
5 Central Spain 1	0.060	**0.107**	0.004	−0.026		-0.016	−0.011	0.009	**0.059**
6 Central Spain 2	**0.071**	**0.120**	0.011	−0.020	−0.021		−0.017	−0.008	**0.102**
7 Eastern Spain	**0.074**	**0.122**	0.011	−0.030	−0.026	−0.029		−0.035	**0.127**
8 Balearic Islands	**0.146**	**0.207**	0.060	0.008	−0.005	−0.016	−0.019		**0.177**
9 Central Ukraine	−0.017	0.005	0.005	0.027	**0.050**	**0.062**	**0.063**	**0.127**	

## Data Availability

Data supporting the conclusions of this article are included in the article. The sequences generated in the present study were submitted to the GenBank database under accession numbers MW438351–MW595608.
